# Insulin Injection Technique Questionnaire: results of an international study comparing Brazil, Latin America and World data

**DOI:** 10.1186/s13098-018-0389-3

**Published:** 2018-11-27

**Authors:** Luis Eduardo Calliari, Laura Cudizio, Balduino Tschiedel, Hermelinda C. Pedrosa, Rosangela Rea, Augusto Pimazoni-Netto, Laurence Hirsch, Kenneth Strauss

**Affiliations:** 1 Pediatric Endocrine Unit, Department of Pediatrics, Santa Casa de Sao Paulo School of Medical Sciences, Sao Paulo, SP Brazil; 2 Pediatric Endocrine Unit, Department of Pediatrics, Santa Casa de Sao Paulo, Sao Paulo, SP Brazil; 3 Institute of Children with Diabetes, Porto Alegre, RS Brazil; 4 Endocrinology Unit and Research Center–FEPECS, Taguatinga Regional Hospital, Secretariat of Health, Brasília, DF Brazil; 5 Diabetes Unit, Endocrinology and Metabolism Service, Federal University of Parana, Curitiba, PR Brazil; 6 Diabetes, Education and Control Group, Kidney Hospital, Federal University of Sao Paulo, Sao Paulo, SP Brazil; 7 VP Medical Affairs, BD Diabetes Care, Franklin Lakes, NJ USA; 8 BD Diabetes Care, Erembodegem, Belgium

**Keywords:** Diabetes mellitus, Insulin, Injections-subcutaneous, Techniques, Syringes, Needles

## Abstract

**Background:**

In 2014–2015, the largest international survey of insulin injection technique in patients with diabetes taking insulin was conducted in 42 countries, totaling 13,289 participants. In Brazil, patients from five public health centers were included. This study aims to evaluate insulin injection technique in Brazilian patients and compare results with Latin America (LatAm) and World data.

**Methods:**

The insulin Injection Technique Questionnaire (ITQ) survey consisted of an initial patient section (questions applied by an experienced nurse), followed by observation of injection technique and examination of the injection sites by the health care professional.

**Results:**

In Brazil, 255 patients were evaluated: 25% had type 1 diabetes mellitus (T1DM) and 75% had T2DM. In this study, 79% of patients injected less than 4 times a day, and 17.3% used insulin pens, compared to 28% in LatAm and 86% worldwide. Syringes were used by 78% of patients in Brazil, compared to 65% in LatAm and 10% globally. Differences in needle length were substantial—nearly 64% in Brazil inject with 8 mm length needle compared to 48% in LatAm and 27% worldwide. Additionally, 48% of patients in Brazil skip doses, 80% reuse pen needles and 57% reuse syringes with 27% having lipohypertrophy by exam.

**Conclusion:**

Brazilian patients use syringes more and pens less, inject with larger needles and have more lipohypertrophy when compared to Latin America and World data. Their re-use of needles and syringes is also high. This study showed that in Brazil, teaching of proper injection technique has to be more widespread, and more intensive during diabetes educational sessions, and the type of delivered supplies must be updated to smaller, shorter needles preferred by patients, in order to facilitate adherence to treatment. From the ITQ, we conclude that there are many aspects of insulin injection technique that may be improved in Brazil.

## Background

According to the 2017 international diabetes federation (IDF) Diabetes Atlas, Brazil has about 12.4 million people with diabetes, the highest number compared to South and Central American countries, and nearly 88,300 children and adolescents with type 1 diabetes, making it the country with the third highest number of children with diabetes in the world [1].

Recent nationwide and regional studies showed that glucose control is out of ideal range in the majority of patients. The strongest association of high HbA1c levels was with low or very low socioeconomic backgrounds [2–4]. Patients with this background are usually less educated in relation to diabetes care. Many researches have demonstrated that improvement in insulin injection technique after educational interventions has significant impact in glucose control and HbA1c levels, besides also lowering total daily dose of insulin [5–8]. The correct technique of re-suspension of cloudy insulin, advice on rotation sites, education on avoiding lipohypertrophy and the correct choice of sites of insulin injection have an impact on insulin pharmacodynamics and consequently on glucose variability and control [9–11]. So, the knowledge about insulin injection techniques in Brazilian patients is of great interest, to understand if it is one of the components leading to the sub-optimal glycemic control found in our population.

In 2014–2015 an international survey of patients taking insulin for at least 6 months was conducted in 42 countries, with a total of 13,289 patients evaluated. The intention was to study many countries to provide a representative global analysis of diabetes care, epidemiologic aspects, variations in injection techniques and its outcomes. The global results were published in 2016 [12, 13], becoming an important source of information and also providing data for new recommendations of insulin injection [14]. As the raw data of the original study is available, it is possible to individualize the results by country. Our aims were: a- to evaluate Brazil´s results of injection techniques in patients with diabetes, and b- to compare these results with Latin America and Worldwide data.

## Methods

In Brazil, a total of 255 patients from five public health centers were included. All centers were specialized in diabetes treatment, serving as secondary and tertiary care from the public national health service, in five Brazilian geographic regions: two from Southeast, one from Mid-west and two from South. There was no financial incentive to enroll [12].

The insulin Injection Technique Questionnaire (ITQ) survey consisted of an initial patient section (applied by an experienced nurse) followed by a second section, performed by the patient’s diabetes educator, nurse or physician, when they observed patient injection technique and carefully examined the injection sites. The technique was considered correct when executed as follows: first gently lift a skinfold, then inject the insulin slowly at a 90° angle to the surface of the skinfold, let the needle remain in the skin for a count of 10 after the plunger is pressed down (when using a pen), withdraw the needle from the skin at the same angle it was inserted, only when the needle is completely removed release the skinfold, and at last dispose of the used needle safely [12, 15]. Detailed information about the development of the survey can be found in the original article [12]. Patient demographic data included age, sex, type of diabetes, years with diabetes, years injecting, and devices used. The key insulin injection parameters selected by our analysis were as follows: current practice (injection device and needle length, number of injections per day, use and characteristics of lifted skinfolds (pinch-up), needle entry angle, site rotation, dwell time of needle under the skin, site inspection by a health care professional, needle reuse, sharps disposal), observed anomalies at injection sites (lipohypertrophy), knowledge about injections (identity of trainer, themes covered in injection training), and disposal habits for used sharps [12, 13].

In this analysis, we report results from Brazil, Latin America, and World data, the latter including those respondents from Brazil, which represent less than 2% of the total survey.

All the participants had diabetes and had been injecting insulin for at least 6 months before taking the survey. Each center applied the questionnaire to roughly 25 patients on a sequential basis as consenting and eligible patients entered the clinic, in order to eliminate any bias in the selection. In a survey of this size, not every question was answered by every patient, and small differences for certain parameters may be statistically significant due to the large numbers.

A total number of 13,289 patients from 42 countries participated, in Latin America were 603 and in Brazil were 255. As this was a global survey, there were questions that were not answered and other were not completely clear, so they were excluded from the analyzed data. When such thing happened, the percentages were calculated according to the data that were considered clearly provided. For that reason, the N may vary from one table to another.

The study was conducted according to Good Clinical Practices and the Helsinki accords, and subject identity was kept confidential at all times. Participants were required to provide verbal consent to participate in the study and were informed that they were not put at risk by the study, and that their care would not be affected. Therefore, ethics committee approval was not generally required, but was obtained whenever specifically requested by a center.

Statistical analysis was IBM SPSS Statistics for Windows software, Version 19 (IBM Corp) was used to perform the data analysis. Descriptive statistics, frequencies, and rankings were calculated. Two-tailed tests were used in all the analyses. Initially, results from each of the 42 countries were analyzed independently, and only when the distributions of key demographic parameters (age, sex, body mass index [BMI], and duration of diabetes) were found to be comparable were all the data pooled into an overall database. The threshold for staying in the model was *P* < .05.

## Results

The sample studied in Brazil, Latin America and World is described in Table 1. From the total of 255 patients in Brazil, 25% of patients had type 1 diabetes mellitus (T1DM) and 73% had type 2 diabetes mellitus (T2DM). Duration of diabetes, years taking insulin, TDD insulin and mean HbA1c were similar in Brazil, Latin America, and worldwide (Table 1).

**Table 1:** Characterization of the patients from Brazil, Latin America and World

Characteristic	Brazil	N Brazil	LatAm	N LatAm	World	N World
T1DM	25%	64	31%	183	21%	2790
T2DM	73%	187	68%	408	41%	5378
Other/Not responded	2%	4	1%	8	38%	5057
Age (years)	53.0 (18.9)	255	49.8 (20.1)	599	51.9 (18.1)	13,225
BMI*	28.2 (6.1)	252	27.9 (6.0)	574	26.6 (6.2)	12,806
Years with DM (years)	15.6 (9.5)	248	13.6 (9.6)	572	13.2 (9.7)	9197
Age at diagnosis (years)	36.7 (17.5)	231	35.9 (18.3)	562	39.9 (7.2)	12,737
Years on pills	14.8 (9.8)	148	13.3 (9.5)	272	8.3 (7.2)	6607
Years on insulin	9.8 (8.5)	234	8.2 (7.9)	515	8.7 (8.9)	8242
TDD regular (Units)	12.2 (9.8)	86	13.9 (9.9)	167	27.0 (20.7)	1422
TDD rapid analogues	17.2 (11.5)	39	18.9 (11.4)	150	31.9 (21.6)	3467
TDD NPH	39.3 (26.8)	219	37.6 (23.8)	391	31.6 (24.4)	1134
TDD basal analogues	34.8 (14.7)	31	26.9 (13.1)	186	27.6 (19.5)	4709
TDD premix	136	1	62.4 (48.3)	7	43.0 (25.3)	1796
Overall TDD	45.9 (30.9)	242	42.5 (26.5)	579	48.5 (32.4)	7756
HbA1c (%)	8.45 (1.8)	163	8.34 (1.8)	460	8.47 (2.1)	7663

*BMI* body max index (calculated as the weight in kilograms divided by the height in meters squared), *DM* diabetes mellitus, *T1DM* type 1 diabetes mellitus, *T2DM* type 2 diabetes mellitus, *TDD* total daily dose, *NPH* neutral protamine Hagedorn, *HbA1c* glycated hemoglobin (%[mmol/mol])

Regarding the number of injections per day, 79% of patients in Brazil injected less than 4 times a day, compared to 54% worldwide, and 78% in Latin America.

The use of devices to deliver insulin by patients from Brazil was markedly different from the rest of the world (Table 2): syringe use was nearly 8 times more common in Brazil than worldwide.

**Table 2:** Devices used to inject insulin in Brazil, Latin America and the World

Device	Brazil	Latin Am	World
Pen	44 (17.26%)	170 (28.24%)	11,070 (86.29%)
Syringe	199 (78.04%)	392 (65.12%)	1238 (9.65%)
Pen and syringe	12 (4.70%)	39 (6.48%)	337 (2.63%)
Pump	0	1 (0.16%)	184 (1.43%)

When questioned about needle length, patients in Brazil were using longer needles than in Latin America and in the world (Table 3). Regarding remix of cloudy insulin, 93% said that they did it, but only 15% reported to roll it 20 times. More importantly, 76% referred to roll 10 times or less.

**Table 3:** Needle length used to inject insulin in Brazil, Latin America and the World

Needle length (mm)	Brazil, N (%)	Latin Am, N (%)	World, N (%)
4	10 (7%)	41 (10%)	2205 (28%)
5	15 (10%)	50 (12%)	1599 (20%)
6	18 (12%)	96 (22%)	1582 (20%)
8	95 (64%)	207 (48%)	2135 (27%)
Other/NA	10 (7%)	34 (8%)	395 (5%)

*NA* non available

The nurses assessed the injection techniques in practice, so the information provided by the patient when filling the questionnaire could be compared with what they do in practice. When asked about rotation of application, 86% of patients responded that they did so, but only 59% of them did it correctly when assessed by the nurse. Worldwide, 83.9% reported rotating their injection sites, and 70.6% did so correctly per nurse inspection.

When questioned if sometimes they skipped doses, 48% reported they did, and 37% skipped them several times a month. Almost one third (33%) said the reason for skipping doses was that they forgot to inject.

Table 4 shows that needle reuse—with both pens and syringes—is common worldwide (56% and 39%), but even more so in Brazil (80% and 57%) and in LatAm (71% and 61%). Saving money and convenience were frequent explanations.

**Table 4:** Needle reuse frequency in Brazil, Latin America and the World

Item	Brasil pen	Brasil syringe	LatAm pen	LatAm syringe	World pen	World syringe
*Reuse needles*	n = 59	n = 209	n = 238	n = 449	n = 11,961	n = 2711
Yes (%)	80	57	71	61	55.80	38.80
No (%)	20	43	29	39	44.20	61.20
*Frequency of reuse*	n = 48	n = 121	n = 191	n = 283	n = 3985	n = 1126
2 times (%)	23	30	31	44	30.7%	35.4
3–5 times (%)	40	45	28	39	39.7	44
6–10 times (%)	21	17	15	11	16	11.4
> 10 times (%)	17	7	15	6	13.60	9.2

When examined by the nurse, 27% had lipohypertrophy (LH) − 39% reported to inject into LH sometimes or always, and 69% justified that they did it because it was a routine. When asked about examination of their injection sites, 55% of the patients said that they could not recall it ever being done, compared to 38.9% worldwide.

Of all the patients, 37% were trained by a diabetes nurse, 31% by a general nurse, and the others don’t remember having been trained. Regarding some specific topics, more than 40% don’t remember to have ever been trained about how long they should keep the needle (44%), how to mix insulin (41%), what is the injection depth (44%) or how long to keep skin fold (49%).

Regarding the time patients maintained the needle under the skin during injection, 25% kept the injection for less than 5 s, 44% between 5 and 10 s, and 26% more than 10 s (5% didn´t know). About skin fold, 70% did it correctly, but only 39% released the skin fold only when the needle is removed.

In Brazil, the initial disposal for sharps (where do you put the used sharp?) was into specially made containers in 38% and home container (such as a used one for laundry detergent) in 38% of the patients, compared to 21% and 23%, respectively, worldwide. Roughly one-fifth (21%) of Brazil patients said they put their used sharp into the trash, with the cap on—less than half of the 48% who reported this, worldwide. The final disposal (what do you do with the waste?) was to take it to the hospital in 74% of the surveys.

## Discussion

Insulin injection technique is part of diabetes education and essential for achieving optimal diabetes control. The ITQ survey involved 13,289 patients from 42 countries and revealed many important aspects of insulin injection all over the world. The amount of data obtained generated important information and was used to revise guidelines in the light of new recommendations [14, 16]. Due to its large size, it is possible to extract data to evaluate individual countries separately, provided the specific number of patients is sufficient. In this study, we wanted to know Brazil-specific data, and also compare its results with other regions. We opted to evaluate Latin America and the World results, so we would have a taste of regional and global practices, and see if they differed from ours.

While there are a number of interesting findings revealed by the ITQ survey, the most striking difference between insulin-taking patients in Brazil and the rest of the world is the nature of the devices that patients use. In Brazil, approximately, only 17% uses insulin pens, compared to 86% worldwide. Syringes, approximately, are used by 78% of injecting patients in Brazil (with a small number using both), compared to 9% globally. Needle length is also notable—nearly 64% in Brazil inject with an 8 mm length needle, compared to 27% worldwide. The 4 mm pen needle, introduced in 2010, is now used by more than one-fifth of injecting patients globally—a notable change—whereas less than 8% do so in Brazil. Most of the world has undergone a major “shift-to-short” in terms of insulin delivery needles, whereas this has lagged in Brazil. The likely reason is that the public health system provides most of the devices used by patients, and usually those devices are syringes with 8 mm needles. The published literature consistently shows favorable findings for use of short needles instead of longer ones, including in obese patients taking large injections of insulin [17–20]—glycemic control is the same, there is no increase in leakage or backflow of insulin from the skin, and pain is reduced. Patients consistently prefer the shorter needles, overall. The risk of inadvertent IM injection is also lowest with the 4 mm length needle (or 6 mm for syringes). Based on this evidence, the post-FITTER Injection Technique Recommendations [14] strongly recommend use of the 4 mm needles as the safest for all patients, including both children and adults, and these have been reflected in the official Brazilian Diabetes Society (SBD) Positioning Statement published online in March 2017 [16].

Most patients in Brazil with T2DM uses NPH and Regular insulin and, on average, spend more years in treatment on oral medicines than the world average. When they are already taking insulin, basal insulin doses (NPH or analogues) are higher than the world average, and the doses of bolus insulin (R and fast analogues) are much lower. Considering that 85.8% of patients use NPH insulin, knowing that only 15% report re-suspending cloudy insulin correctly is alarming. The recommendations suggest performing at least 20 soft movements, and not doing it properly may influence the absorption and action of the injected insulin, substantially [9–11].

As for the number of injections/day, in Brazil 21% make 4 or more injections/day while in the world 46% do so. In a large previous study in Brazil, from the 5750 patients with Type 2 diabetes, 35% injected insulin and 58% of those did 2 injections/day [3, 4]. This suggests that we have more difficulty in establishing intensive insulin therapy, which is currently recommended for patients DM1 or DM2 (when indicated). Mean HbA1c in Brazil, Latin America and the world is still far from the 7% value related to a lower risk of progression of microvascular damage. The attainment of this target of treatment is achieved far more effectively with intensive treatment [21].

The assessment of the injection technique is also very relevant in this study, since the information filled out by the patient in the questionnaire was confirmed with the observation of the nurses in practice. The difference between what the patient “thinks he does” and what he “actually does” shows how important it is to review the technique regularly and not only briefly question it theoretically. The main protective factor for the development of lipohypertrophy is the proper rotation of application sites or regions [22, 23]. In the questionnaire, 86% of the patients referred to doing the rotation, but according to the nurse only 59% did it correctly. Correct site rotation involves moving from one injection to the next by at least one finger-width (about 1 cm) in a pattern such as a line, or a circle, or a square within a region, and *between* regions as well [13, 14]. The main reasons for incorrect rotation, according to other studies, could be not only the lack of information, but also the assumption that lipohypertrophic areas are less painful and also rooted habits [24]. As for the skin fold, which is very important when using needles longer than 5 mm [25], 70% of the patients did the pinch correctly, but only 39% removed the needle before releasing the skin fold.

A major fact in this aspect is that more than 40% of patients report never having received guidance on how long they should keep the needle on the skin after the application, how deep the needle should penetrate, how long they should keep the fold.

Results showed that reuse of needles and syringes are higher in Brazil than in other parts of the world, mainly due to costs and convenience. Interestingly, the reuse of pen needles is higher than syringes, probably due to government supply of syringes, but not pen needles. Important to mention that there is a current debate, confronting the positions of the Ministry of Health, allowing the reuse of needles and the Brazilian Diabetes Society, which recommends no reuse [16, 26]. The main issue, costs aside, is that although patients can reuse needles and do not get local infections or abscesses, the needles do become duller, and using needles by roughly 5 times or more is strongly associated with prevalence of lipohypertrophy [13, 14].

Regarding lipohypertrophy (LH), nearly 32% said they “have lumps at injection sites that have been present for weeks, months, or years”, and 27% presented a lump at one or more of the injection sites on physical examination. The presence of LH is mainly related to incorrect rotation of sites, but also to the frequency of reuse of needles and therapy with insulin for longer periods [13, 22–24, 27]. The presence of LH is associated with higher glycated hemoglobin values (up to 0.5% higher) and higher total daily insulin doses (5–15 U/day) [5, 6, 8, 22, 23, 27]. Intervention with intensive educational measures regarding insulin delivery techniques has shown a reduction in LH, glycated hemoglobin and daily insulin doses, thus promoting improved quality of life for the patient, therapeutic efficacy and lower costs related to insulin therapy and chronic complications due to poor glycemic control, although there are limitations to each of these reports [5, 6, 8, 22, 23, 27].

The information about sharps disposal seems to be clearer, though. While in the world about 48% of patients put used needles (recapped) in the common garbage, according to ITQ in Brazil more than 76% is discarded in appropriate containers at home and at health services, which are committed to properly dispose of waste. In another Brazilian study, in Ceará, 50% of the patients reported having received guidance about sharps disposal, but 57.1% did so in common trash [28]. These discrepancies could be explained by the fact that this study was done in reference centers, and also because the distribution of proper containers by the government is variable, results depending on which State of the Federation the survey was performed.

Brazil is a country with continental dimensions, and therefore presents particularities when the demographic profile is evaluated. In this study, the five participating centers were from predominantly urbanized regions and referenced care for diabetes treatment. It is possible that the national scenario is even less favorable in some respects, similar to the ITQ data presented in India [29], where primary care and insulin delivery guidelines are also performed by general practitioners in very quick consultations, without the support of nurses specialized in diabetes and having only the medications and supplies available in the public health system.

The characteristic of the Brazilian health system has also unique characteristics, difficult to balance in the development of a study like this one. About 24% of the Brazilian population relies on private healthcare, varying from 5% to more than 70% of coverage depending on the state [30]. There is evidence that the patients who receive diabetes medical care at private services have lower HbA1c than those who depends of the public health system [4]. Interestingly, in this survey we found similar HbA1c in Brazil, Latin America and the World, regardless of all the differences found in injection technique, type of insulin, devices used and total insulin daily dose. This is in accordance with the known difficulties patients with diabetes have in obtaining a good metabolic control all over the globe [1, 4, 21]. The public health system provides NPH and Regular insulin for free, and needles, syringes, lancets and reagent strips for blood glucose monitoring. In special cases some patients also receive free analog insulin, pens and needles for pens for application. Generally speaking, most patients uses what is provided by the state, while those who have some financial capacity buy their own medicines and supplies and often can choose between spending with better insulin or with pen needles. This shows that the choice of insulin and the devices may not be what the doctor considers best for that patient specifically, but what he or she has available for use according to the health system and the patient’s purchasing power.

The limitation of this study is related to the fact that the participating centers may not reflect the reality of all the regions of the country. The strengths are the large number of patients evaluated, the appraisal about many aspects of insulin treatment and the possibility to compare different countries within the same protocol. Its results can contribute to the comprehension of the care of diabetes in the country, helping the development of better health policies.

## Conclusions

Data from ITQ Brazil allow us to conclude that there are many aspects of insulin injection that need to be improved. Brazilian patients are using more syringes and less pens, are being exposed to larger needles and are having more lipohypertrophy when compared to Latin America and World data. Their re-use of needles and syringes is also higher than worldwide. After the ITQ was published, the Brazilian Diabetes Society proposed a new guideline about insulin injections [16], and the information is being spread by many kinds of media. The ITQ study showed that, in Brazil, teaching of proper injection techniques has to be more intensively widespread during diabetes educational sessions, and the delivery of supplies updated, in order to increase adherence to treatment.

## Abbreviations

BMI: body max index
DM: diabetes mellitus
HbA1c: glycated hemoglobin
ITQ: Insulin Injection Technique Questionnaire
LatAm: Latin America
LH: lipohypertrophy
NPH: neutral protamine Hagedorn
T1DM: type 1 diabetes mellitus
T2DM: type 2 diabetes mellitus
TDD: total daily dose
